# Advanced Applications of Robotics in Digestive Surgery

**Published:** 2011-10-17

**Authors:** Alberto Patriti, Pietro Addeo, Nicolas Buchs, Luciano Casciola, Philippe Morel

**Affiliations:** * Department of Surgery - Division of General, Minimally Invasive and Robotic Surgery - ASL 3 Umbria, Hospital San Matteo degli Infermi ,Spoleto, Italy; ° Pole des Pathologie Digestive, Hépatiques et de la Transplantation, Hôpital de Hautpierre, Hôpitaux Universitaires de Strasbourg, Université de Strasbourg, France.; § Clinic for Visceral Surgery and Transplantation, Department of Surgery, University Hospitals of Geneva, Switzerland

**Keywords:** robotic surgery, stomach, liver, pancreas, rectum, cancer

## Abstract

Laparoscopy is widely recognized as feasible and safe approach to many oncologic and benign digestive conditions and is associated with an improved early outcome. Robotic surgery promises to overcome intrinsic limitations of laparoscopic surgery by a three-dimensional view and wristed instruments widening indications for a minimally invasive approach.

To date, the more interesting applications of robotic surgery are those operations restricted to one abdominal quadrant and requiring a fine dissection and digestive reconstruction.

While robot-assisted rectal and gastric surgery are becoming well-accepted options among the surgical community, applications of robotics in hepato-biliary and pancreatic surgery are still debated.

## Introduction

Robotic surgery is evolving as a therapeutic tool for cardiac and urological applications. [1]

However, its role in advanced digestive procedures has not been well defined. Multiple institutions have published their early experience with robotic technology for general surgery procedures, demonstrating feasibility and safety. [2] Several authors have found no added benefit of robotic technology over laparoscopic techniques in simple digestive surgery procedures, such as Nissen fundoplication or splenectomy. [3, 4]

Conversely, benefits of robotics seem more evident where a fine dissection and complex surgical reconstructions are required. [2]

A common feature of the procedures that are commonly carried out robotically is a fixed operating field (i.e. rectum, pancreas, stomach, liver). The latter aspect can be relied to the characteristics of the unique robotic system available to date, the Da Vinci (Intuitive, Sunnyvale, CA). Main aspects of this system are a 3D high definition vision and endowrist instruments allowing for enhanced dexterity, precision and control. The arms are installed in a cart that is docked close to the patient table. Actually, the cart is heavy and once the robotic arms are connected to the trocars changing position and operating field is relatively challenging.

Herein, we reviewed the current literature describing the most promising applications of the Da Vinci robotic system (Intuitive, SunnyVale, CA) in advanced digestive surgery.

## Gastric surgery

### Bariatric surgery

Morbid obesity has become a major health issue in developed countries. For many years now, robotics has been shown as a valid option in the armamentarium of bariatric surgery.

Many groups have reported their results of robotic Roux-en-Y gastric bypass (RYGB). [5–8]

Overall, the robotic results compared favorably to laparoscopic RYGB. Interestingly, the operative time has been reported shorter with the robotic approach in several series [9] even during the learning curve [10]. Yet, these results were not reproduced by other teams. [6, 11, 12]

In terms of postoperative outcomes, Snyder et al [5] in one of the larger series reported a lower morbidity rate after robotic RYGB when compared to laparoscopy. Indeed, after a comparison of 320 robotic vs. 356 laparoscopic RYGB, they found a reduced gastrointestinal leak rate (0 vs. 1.7%; p=0.05). More recently, Hagen et al [13] have published a large comparison of 524 open, 323 laparoscopic and 143 robotic RYGB. They have shown a significant reduction of anastomotic leak after open or robotic approach (1.9% and 0% respectively) when compared to laparoscopic approach (4%). Moreover, in this study, robotics has been reported cost effective by avoiding such complications: robotic USD 19,363, laparoscopic USD 21,697 and open USD 23,000.

The experience of robotic adjustable gastric band (AGB) is still limited. [14–16]

Recently, Edelson et al [17] reported a large series of robotic AGB. They compared 287 robotic to 120 laparoscopic AGB. While robotic and conventional approaches were similar in terms of complication rates, operating time, and length of postoperative hospital stay, Edelson et al [17] found a significant reduced operative time with the robotic approach for patients with a BMI >50 kg/m^2^. However, others failed to show similar results in children and adolescents. [18] The exact role and improvement of a robotic approach is not yet clear and should be reserved for difficult cases or redo cases, and probably in the future for robotic single site surgery.

Concerning robotic sleeve gastrectomy and duodenal switch, the data remains scarce. Few groups published their experience. [19–23] If overall they reported the feasibility and safety of the procedures, the real interest of robotics for simple sleeve gastrectomy remains questionable. Ayloo et al [19] compared 30 robot-assisted to 39 laparoscopic sleeve gastrectomies and found no differences in terms of outcomes. The robotic group showed a longer operative time (+21 minutes; p=0.003), due to the staple line that was oversewn in the robotic arm and not in the laparoscopic arm. However, for duodenal switch, the robotic technology might be a real help during the duodenoileostomy [20].

### Oncological resections

Laparoscopic gastrectomy has been reported not only feasible but also safe with good oncological outcomes [24] and even better early postoperative outcomes when compared to open. [24–26] Recently, robotics has been developed to overcome the potential limitations of laparoscopy that could render a precise lymphadenectomy difficult [27]. Moreover, wedge or atypical resections can be managed by a robotic approach, as it was reported for gastrointestinal stromal tumor [28].

Since 2003, several studies have been published, reporting encouraging results after robotic gastrectomy (**Table1**). [2, 29–38] In a recent systematic review, Buchs et al [39] reported 199 patients who underwent a robotic gastrectomy for cancer. They reported a mean operative time of 265 and 334 minutes for total and subtotal gastrectomy respectively. The mean number of lymph nodes harvested was 32, with a mean estimated blood loss of 113 ml. Only 2.5% of patients were converted. The perioperative mortality was low (1.5%) and the complications rate was acceptable (14.6%). Overall, the mean hospital stay was 10 days. These results were at least comparable if not better than those reported for an open and laparoscopic approach. [24, 26, 40–42]

Different groups showed that a robotic approach could be carried out with less blood loss when compared to laparoscopic or open group. [31, 43] The same is true concerning the hospital stay [44]. Moreover, the conversion rate could be lowered by a robotic approach [39]. However, these good results are reported at the cost of a longer operative time [2] and probably a higher financial cost even if it was only poorly evaluated [27].

From an oncological point of view, the number of harvested lymph nodes complied with Western criteria of a minimum of 25 retrieved nodes [45]. However, it remains true that long term data is not yet available [39].

In 2011, the learning curve of robotic gastrectomy was assessed by a Korean team [46]. They found that surgeons with sufficient experience in laparoscopic gastrectomy (between 68 and 400 cases in this study) can rapidly overcome the learning curve for robotic gastric surgery, reported in between 6 and 18 cases. Of note, the specific learning curve for laparoscopic gastrectomy was shown to be more than 60 cases [47]. However, other teams believe that the bridge between open and robotic surgery does not necessarily require the experience of laparoscopy, as it was demonstrated for hepatobiliary surgery. [48–50]

In the largest series so far, Woo et al [38] performed 236 robotic gastrectomies with encouraging results in a high volume center. Moreover, they compared retrospectively this data to 591 laparoscopic gastrectomies during a period of 6 years. They found a longer operative time associated with a robotic approach (+49 minutes). On the other hand, they reported a reduced blood loss (91.6 ml vs. 147.9 ml; p=0.002). If there was no statistically significant difference in terms of morbidity, there was a positive trend in favor of robotics (11% vs. 13.7%; p=0.3). Recently, Patriti et al [34] assessed the role of robotics for the treatment of cardia carcinoma. They reported 17 resections, among them 14 extended gastrectomies, 2 transhiatal distal oesophagectomies and one transthoracic distal oesophagectomy with proximal gastric resection. The results were satisfying and the oncological follow up showed 4 recurrences (2 multivisceral, one pulmonary and one nodal) after a mean of 20 months. While preliminary, this data supports the use of robotics for cardia tumors. Finally, several studies suggest that the robotic approach is a valid alternative to laparoscopic surgery with benefits for the patients in terms of perioperative outcomes. The results reported by experienced centers are encouraging and the oncological rules were respected. However, it is probably still too early to recommend a routine use of da Vinci system for oncological resections, and randomized controlled trials with long term follow up are really needed.

## Hepato-biliary surgery

Laparoscopic hepatic resections (LHR) are considered to be as safe option for the treatment of benign and malignant nodules of the liver (OHR). Clear margins can be maintained and the postoperative course is generally better tolerated than after OHR. [51]

Nevertheless, it is difficult to reproduce laparoscopically elementary maneuvers of open surgery - i.e. intermittent pedicle clamping, knots and sutures – making demanding hemorrhage control and bile duct reconstructions. [52]

An international consensus conference was organized to evaluate the status of LHR. [53] According to this consensus report, the best indications for laparoscopic liver resection were solitary lesions, 5 cm or less, located in the peripheral liver segments (segments 2–6), whereas lesions adjacent to major vessels, near the liver hilum or in segments 1,7 and 8 were not considered suitable for LHR because of the potential risk of massive bleeding and the potential need for biliary reconstruction.

Robotics promises to overcome these limitations providing a greater maneuverability and vision than traditional laparoscopy.

Interests in robotic liver resections dates back 2008 when Choi et al. published their first series of four liver left lobectomies. [54]

After this report more than ten case series from Asian, European and American institutions were published focusing mainly on the feasibility of robotic liver resections for lesions located in the anterior segments (from 2 to 6) **(table 2)**. [49, 55–63]

Berber et al. prospectively compared nine patients who underwent robotic resection of peripherally located malignant lesions with 23 matched patients who underwent laparoscopic resection at the same institution. The authors did not find significant differences between the two techniques even though robotic resection appeared to be more precise and blood-less. [64]

A conceivable explanation to these results can be relied to the selection of patients with a tumor in the peripherally located liver segments. For easily accessible lesions or when there is not a vascular involvement laparoscopy and robotics seem to be equivalent in achieve a margin free, safe liver resection. On the contrary, potential benefits of robotics in LHR are highlighted approaching lesions located in the postero-superior segments or involving vascular structures and when a complex reconstructive phase is required. Casciola et al. demonstrated that robotics allows an easy access to the postero-superior segments to carry out parenchymal-saving resections instead of a major hepatectomy even when the tumor is in close contact with a great liver vessel. [61]

The use of endo-wristed instruments for parenchymal transection was the main technical tip evidenced in this study. Reproducing a traditional Kelly-clamp crushing technique for curved resection planes made possible to perform liver resections with a maximal parenchymal preservation even for lesions deeply located and in contact with the main liver vessels.

This technique is not easily reproducible in laparoscopic surgery favoring the use of the harmonic scalpel, the dissecting sealer and other transection devices that force to follow straight resection lines.

Giulianotti et al. recently described the applications of robotics in major liver surgery. [49, 65, 66]

A total of 24 right hepatectomy were carried out by a single surgeon with zero mortality and with a low conversion rate (4.2%). Post-operative morbidity was minimal (25%) as well as blood loss. After a mean follow-up of 34 months no port-site metastases were described among the oncologic cases. In the same center the Da Vinci robotic system was used to complete complex biliary reconstructions with curative and palliative intent. [48, 57]

These series show that robotic surgery could offer significant advantages over laparoscopy. Specifically, the da Vinci robotic system enables the surgeon to have 3D stereoscopic visualization, intuitive finger-controlled movements, and EndoWrist technology. This translates into more careful, precise dissection of fine structures and superior dexterity in knot tying and suturing.

Finally, possible advantages of robotics in hepato-biliary surgery are going to be delineated but prospective controlled studies involving a large number of patients are required for definitive results.

## Pancreatic surgery

Despite recent progress in chemotherapy regimens, surgery still represents the best treatment for many types of pancreatic tumors. Pancreatic surgery represents one of the most challenging areas in the field of digestive surgery. Surgical approaches to the pancreatic gland require a detailed knowledge of the regional anatomy, dedicated surgical training and adequate skills to dissect tissues and vessels correctly and to perform digestive reconstruction [67]. Pancreatic surgery has been historically associated with high postoperative morbidity and mortality^2^. Adequate exposure of the pancreatic gland is usually obtained through a large abdominal incision, which is among the possible causes of postoperative morbidity. To reduce the morbidity rates associated with open pancreatectomies, there has been growing interest in recent years in minimally invasive laparoscopic pancreatic surgery. However the development of laparoscopic pancreatic surgery has been slow compared to other fields of abdominal surgery. Reasons for that are multifactorial and include the technical challenges of open pancreatic surgery, the intrinsic technical limitations of laparoscopy, the fear of increased morbidity, oncologic concerns, and slow acceptance of laparoscopy among pancreatic surgeons [68]. Currently most of the laparoscopic pancreatic resections performed are represented by distal pancreatectomy (DP) owing to the relative ease in achieving exposure and the absence of digestive reconstruction required. The laparoscopic approach for DP offers all the benefits of minimally invasive surgery for patients, including decreased postoperative pain, earlier recovery and prompt return to daily activities [69]. On the other hand pancreatic resections for pancreatic head and neck tumors have been not widely performed since they require an extensive dissection, followed by a complex digestive reconstruction, which remains difficult to perform laparoscopically because of the restricted 2-dimensional view and the limited degree of freedom of surgical instruments [70]. To overcome the present technical limitations of laparoscopic surgery, robotics has been added into the technical armamentarium of pancreatic surgeons. The first robotic pancreatic resection was published in 2003 by Giulianotti et al [2] in Europe and included 16 pancreatic resections. In the USA in the same year Melvin et al [71] published a case report of a robotic resection of a pancreatic neuroendocrine tumor. Since that time, the application of robotics for pancreatic surgery has been poorly reported until 2010 when a large series of pancreatic resection has been published^8^. Until June 2011, excluding some case reports published, about 235 cases of robotic pancreatic resection have been described^9–19^ (Table 3). The experience is in an early phase and questions about the safety, feasibility, potential advantages and cost effectiveness of robotic pancreatic surgery remain opened.

With regard to the safety and feasibility, the analysis of the data reported shows an overall conversion rate averaging between 0% and 37.5%, a mortality rate of 2.12 % and a morbidity rate in the range between 0 and 60% according to the specific type of resection performed. It is noteworthy to note that all these series have been reported in the last two years and 121 (51.4%) of the robotic resections reported were pancreaticoduodenectomies (PD). A recent review of the literature found a total of 285 laparoscopic PDs published over a larger period of time [72]. Therefore the first consideration is that robotics seems to find its specific application in the field of pancreatic surgery for resections that combine complex dissection and reconstruction. From a technical point of view robotic could improve certain steps of PD such as lymhadenectomy and the uncinate process dissection. In addition the microsurgical ability provided by the robotic system could confer superior dexterity when performing biliary, pancreatic and even vascular reconstruction [73]. For PD, the average operative time reported by most of the authors is slightly longer than the open counterpart probably reflecting a lack of specific experience with this operation. Morbidity and mortality compare favorably with that of open PD, with one study reporting good outcomes also in fragile patients older than 70 years. [48] Pancreatic fistula remains the most common complication with an incidence similar to the open counterpart. Even if performed by a minimally invasive approach, robotic PD achieves a postoperative length of stay similar to open surgery reflecting the specific complications of this operation which often requires interventional management and prolonged postoperative length of stay. Concerning the type of pathology selected for the robotic approach, periampullary malignancies are the majority of those with half of the cases represented by pancreatic adenocarcinomas (PAC). Giulianotti et al^8^ reported 26 robotic PDs for PAC with 9 patients alive and disease free at a mean follow-up time of 16.8 months (8–47 months) and no port-site recurrences. Furthermore, the analysis of the series reported for robotic PD showed that the mean number of lymph node retrieved and the rate of positive-margin appear comparable to those reported for open PD. However the number of reported cases remains too low to draw firm conclusion. Up to date, only two studies have compared the robotic and the conventional approach for pancreatic surgery. Zhou et al [74] in a short series of 16 PDs (8 robotic and 8 open) reported similar R0 resection rate (87.5%vs 100%,P=0.005), increased operative time (718± 186 vs 420± 127 min, P=0.011), decreased intraoperative blood loss (153±43 vs 210±53ml,P=0.045), complication rate (25% vs 75%,P=0.05), and length of postoperative stay for the robotic approach(16.4 ± 4.1 vs 24.3 ± 7.1 days, P=0.04). The decrease in intraoperative blood loss in the robotic group could be related to the more delicate management of pancreatic uncinate process and its venous connections that often cause sustained bleeding in open PD. Waters et al [75] analyzed a cohort of 77 distal pancreatectomy (32 open, 28 laparoscopic and 17 robotic) comparing three different approach for DP. Indications for surgery were more frequently represented by low malignant tumors in the robotic and laparoscopic groups and PAC in the open group. Spleen preservation occurred in 65% robotic distal pancreatectomies versus 12% and 29% in open distal pancreatectomies and laparoscopic distal pancreatectomies (P < .05). The robotic group showed a statistically significantly longer operative time (P<.05) and shorter length of stay (P=0.04). The total cost was $10,588 in robotic distal pancreatectomies versus $16,059 and $12,986 in open distal pancreatectomies and laparoscopic distal pancreatectomies with no differences between the three groups. Beyond the clear advantages in the reduction of the postoperative length of stay, relevant findings emerging from this study are the increased rate of spleen preservation and its cost effectiveness. Spleen and splenic vessels preservation during distal pancreatectomy can be particularly challenging by a minimally invasive approach due to the necessity to divide all the vascular connections of the distal pancreas from the splenic vessels. Robotics probably improves the dissection of these structures because of its magnified and stable view and instruments with wide range of motion, however a significant selection bias is present in this study and prospective validations of this finding seem necessaries. The similar cost between the tree approaches is motivated by a significant reduction in the mean postoperative length of stay, in the rate of major morbidity and by the decreased manipulation and torque of the transabdominal port in the robotic group that reduce the overall morbidity compared with laparoscopy and resulted in the cost effectiveness of the procedures.

In conclusion current available data shows safety and feasibility of robotic pancreatic surgery. Specific perceived technical advantages of this approach are an increased dexterity for performance of reconstruction during PD and splenic vessels preservation during DP.

Cost effectiveness and potential advantages over open and laparoscopic surgery need a validation in large prospective studies.

## Rectal surgery

Laparoscopic anterior resection with total mesorectal excision (TME) is considered a safe treatment option for rectal cancer. [76][76, 77]

Robotic surgery is considered an evolution of traditional laparoscopy improving surgeon dexterity where fine manipulation of tissues in a close, fixed operating field and when hand-sewn sutures and knot tying are required. [2, 78]

Therefore rectal surgery fits well with intrinsic characteristics of this device because of the narrow space of the human pelvis and makes the robot especially suitable for the total mesorectal excision (TME).

Two techniques of anterior rectal resection (ARR) are described: a totally robotic anterior resection (TRAR) and a hybrid technique (HT). Common feature of the two techniques is the robot-assisted TME. The HT comprises laparoscopic mobilization of the left colon and a robotic mesorectal excision. [79, 80]

Two types of TRAR are described. In 2004 D’Annibale A. et al. described the technique of TRAR using the three-arm Da Vinci. The procedure entails two steps for robotic cart placement, one for left colon mobilization and one for TME. [81]

Later, two authors independently gave the description of a technique that did not require robot repositioning and is carried out with the aid of the four-arm Da Vinci S robotic system. [82, 83]

In both TRAR and HT, once the TME is completed the assistant divides the distal rectum using a linear stapler through a 12-mm laparoscopic port inserted in the right lower quadrant.

The specimen is extracted through a suprapubic or left lower quadrant minilaparotomy and the stapled anastomosis is carried out trans-anally.

Irrespectively to the type of technique, several comparative studies investigated safety and feasibility of robotic rectal resection in comparison to the laparoscopic and open approach.

Spinoglio et al. reported longer operative times for the robotic arm of his comparative study. The main limit of the study is that the authors compared two series including both colon and rectal resections and no mention is provided about operative time of rectal resections. [84]

In the study from the Hospital San Matteo degli Infermi longer operative times are reported for robot-assisted partial mesorectal excisions (PME) performed for high rectal tumors. However, when a TME is carried out for low and ultra-low rectal tumors an advantage in term of time sparing is highlighted. [85]

Intraoperative complication rate seems to be similar between the two approaches even though a trend toward a less conversion rate during robot-assisted surgery is delineating. In a multicentric study describing the larger series of robot-assisted rectal resection ever published, the rate of conversion is 4.9% and a significantly less conversion rate is also reported in the robotic arm of the comparative study carried out by Patriti et al.. [85, 86]

Subjective surgeon experience is generally considered excellent, especially concerning the precise dissection during mesorectal excision, and the robot is deemed useful also for inferior mesenteric artery dissection and splenic flexure take-down. [2, 87–89]

Pigazzi et al. addressed to the robot also a reduced fatigue probably due to the comfortable position adopted by the console surgeon. [80]

The lack of a tactile feed-back was not felt as a limitation of the robotic system by all authors. [2]

Two papers were specifically designed to investigate the appropriateness of the specimen.

Baik et al. noted that the macroscopic grading of the specimen was complete in 17 out of 18 rectal resections and that this value was significantly higher than that of the laparoscopic arm of their study. [90]

The circumferential resection margin (CRM) was clear with a distance between the tumor and the fascia mesorectalis ranging from 0.1 to 4.5 cm in 142 out of 143 patients. [86]

The mean number of harvested node compares favorably with that of the current literature in all the reports. [85, 86, 90]

Complications after robotic rectal resection are low with a rate of anastomotic failure ranging from 4.8 to10.5%, which compares favorably with that reported in previous large series of laparoscopic rectal resection. [76, 77, 91, 92]

In 143 patients the overall complication rate was 41.3%. [86]

Even though length of hospital stay differs between USA, Europe and Asia due to distinctive health systems, a trend toward a shorter hospitalization is reported and in one study a significant reduction of hospital stay in respect to laparoscopic surgery was reached. [86, 88]

To date, the longer follow-up time is that of the multicentric study involving two European and one US centers. The 3-year disease-free and overall survival rate were 77.6% and 97%, respectively.

Encouraging data emerge from analysis of recurrence sites. Port site and isolated local recurrences were not identified. Local recurrence combined with liver and/or lung metastases occurred in two out of thirteen recurrent patients (1.5%). Distant only metastases occurred in eleven patients (7.7%).

In only one study sexual dysfunction is considered in long-term outcome analysis. Compared to a laparoscopic series no differences were reported in erectile dysfunction, even though the two groups were not matched for tumor location because of a higher number of low rectal tumors operated on with a robotic approach. [85]

Therefore, despite the impressive subjective experience of the surgeon at the console, a few data demonstrates a real impact of this technology on patient outcome. [78] Furthermore, the majority of studies aiming to evaluate feasibility and outcome of robotic rectal surgery enrolled a small number of cases with a short follow-up period [88]. [80, 83, 85]^16, 17, 22, 25^

In conclusion, despite the limitations of the current studies in medical literature, robotics is likely to improve laparoscopic mesorectal excision. Prospective controlled trials should be aimed to verify whether robotic surgery could improve local control of rectal cancer giving to patients a survival advantage and a lower post-operative morbidity rate.

## Conclusions

Robotic surgery is safe and feasible for a variety of advanced digestive surgical procedures. This technology may widen the applications of minimally invasive surgery in the treatment of digestive cancers requiring complex surgeries. However, more clinical experience and further investigation are needed to determine improvement in quality of life and long-term survival.

Prospectively and retrospectively collected data from large series with a long-term follow-up are available for gastric and rectal surgery with encouraging results. In hepato-biliary and pancreatic surgery data available are adequate to demonstrate feasibility and safety of a robotic approach.

Probably, in the forthcoming prospective studies correct variables have to be identified to demonstrate a real advantage of robotic surgery over laparoscopy. Robotics is likely to be considered another form of minimally invasive surgery. Consequently, conventional variables, such as length of hospital stay, postoperative pain and incision lengths, result not significantly different in comparison to laparoscopic operations.

## Figures and Tables

**Table 1.** Experience of robotic gastrectomy reported in the current literature including more than 10 cases.

Authors	Year	n	Operative time in minutes	EBL in ml	Conversion	Complication	Mortality	Lymph nodes	Hospital stay in days
Giulianotti et al.^1^	2003	21	350/365*	NA	4.8%	19%	4.8%	NA	10
Patriti et al.^31^	2008	13	294.6/282*	103	NA	46%	0	28.1	11.2
Song et al.^2^	2009	100	231.3***	128.2	0	13%	1%	36.7	7.8
Tomulescu et al.^35^	2009	12	187/265*	NA	8.3%	NA	0	NA	12.3
Guzman et al.^3^	2009	16	415**	250	6.25%	25%	0	26.8	7
Kim et al.^4^	2010	16	259.2**	30.3	0	0	0	41.1	5.1
Pugliese et al.^5^	2010	18	344**	90	NA	NA	NA	25	10
Park et al.^6^	2011	60	247.3	NA	0	10%	0	NA	6.9
Lee et al.^7^	2011	12	253.7**	135.8	0	8.3%	0	46	6.6
Woo et al.^8^	2011	236	219.5***	91.6	0	11%	0.4%	39	7.7
D’Annibale et al.^25^	2011	24	267.5***	30	NA	8%	0	28	6
Patriti et al.^9^	2011	17****	327.2	279	0	41.1%	0	28	12

N: number of patients. EBL: estimated blood loss in ml. NA: not available.

*: for total and subtotal gastrectomy respectively.

**: only for subtotal gastrectomy.

***: including subtotal and total gastrectomy.

****: including 3 oesophaectomies for cardia carcinoma.

**Table 2:** Reported series of robotic liver surgery

**Author**	**Year**	**Country**	**Series**	**Surgery**	**Conclusions**
**Choi**	*2008*	*Korea*	*4*	*Left lobectomy*	*Feasibile* *Early recovery*
**Vasile**	*2009*	*Romania*	*1*	*Left lobectomy*	*Feasible*
**Patriti**	*2009*	*Italy*	*7*	*Anatomical and non-anatomical resections + colectomy*	*Feasible*
**Giulianotti**	*2010*	*USA*	*1*	*Right hepatectomy*	*Feasible*
**Giulianotti**	*2010*	*USA*	*1*	*Extended right hepatectomy*	*Feasible*
**Giulianotti**	*2010*	*USA/Italy*	*70*	*Anatomical and non-anatomical resections*	*Feasible*
**Ji**	*2010*	*China*	*13*	*Major hepatectomies*	*Feasible*
**Berber**	*2010*	*USA*	*9 (robot)* *23 (VL)*	*Minor resections and left lobectomy for peripheral lesions*	*Same outcome*
**Chan**	*2011*	*China*	*27* *16*	- *Minor resections and left lobectomy* *- Bile duct exploration*	*Feasible*
**Wakabayashi**	*2011*	*Japan*	*4* *2*	-*Minor resections and left lobectomy* *- Bile duct exploration*	*Feasible*
**Sugimoto**	*2011*	*Japan*	*4* *4*	-*Left lobectomy* *- Cholecystectomy*	*Feasible even with a single port*
**Casciola**	*2011*	*Italy*	*23*	*- Minor and major resections in all liver segments*	*Parenchymal preservation feasbile even in postero-lateral segments*
**Giulianotti**	*2011*	*Italy/USA*	*24*	*Right Hepatectomy*	*Feasible*

**Table 3:** Reported series of robotic pancreatic surgery

**Author/Year**	**N**	**Type of resection**	**Approach**	**OR time (min)**	**EBL (ml)**	**Conversion (%)**	**Mortality (%)**	**Morbidity (%)**	**Length of stay (days)**
**Giulianotti PC 2010**	134	PD(60), DP (46),CP(3), TP(1), En(3), Other(21)	R	331 (75–660)	NA	10.44%	2.23	26%	9.3 (3–85)
**Kendrick ML 2010**	65	PD	57L/8HR	368 (258–608)	240 ml	4.6%	1.6%	42%	7 (4–69)
**Narula VK 2010**	8	PD	HR	420 (360–510)	0	37.5%	0	0	9.6
**Chan OCY 2011**	12	PD (8), DP (2), Other (2)	10R/2H	478 (270–692)	200 (30–300)	8.3%	0	33%	12(6–21)
**Kang CM 2010**	5	CP	3R/2H	480 (360–480)	200 (100–600)	0	0	NA	14.6±7.7
**Horiguchi A 2011**	3	PD	3 R	703±141	118 ± 72	0	0	33 %	26±12
**Waters JA 2010**	17	DP	R	298(191–418)	270 (20–1200)	12%	0	18%	3.8
**Zhou NX 2011**	8	PD	R	718±186	153 ± 43	0	0	25%	16.4±4.1
**Zureikat AH 2010**	30	PD (24), CP (4), Other (2)	R	512 (327–848)	320 (50–1000)	0	3.3%	50%	9(4-87)
**Giulianotti PC 2011**	5	TP	R	456 ± 96	310 ± 50	0	0	40%	7±2
**Giulianotti PC 2011**	5	EP-VR PD(2), DP(3)	R	392± 66	200 ± 61	0	0	60%	11± 2

**PD=pancreaticoduodenectomy;DP=distal pancreatectomy;CP=central pancreatectomy;Other: other type of resection including digestive derivation for chronic pancreatitis; TP=total pancreatectomy; En=enucleation; EP-VR= extended pancreatectomy with vascular resection. R=robotic; L=laparoscopic; H=Hybrid; OR=operating room time; EBL=estimated blood loss; NA=not available.**
